# Mutilating Procedures, Management Practices, and Housing Conditions That May Affect the Welfare of Farm Animals: Implications for Welfare Research

**DOI:** 10.3390/ani7020012

**Published:** 2017-02-21

**Authors:** Rebecca E. Nordquist, Franz Josef van der Staay, Frank J. C. M. van Eerdenburg, Francisca C. Velkers, Lisa Fijn, Saskia S. Arndt

**Affiliations:** 1 Behavior & Welfare Group (Formerly Emotion & Cognition Group), Department of Farm Animal Health, Faculty of Veterinary Medicine, University Utrecht, Utrecht 3584CL, The Netherlands; f.j.vanderstaay@uu.nl (F.J.v.d.S.); lisa.fijn@gmail.com (L.F.); 2 Brain Center Rudolf Magnus, Universiteitsweg 100, Utrecht 3584CG, The Netherlands; 3 Department of Farm Animal Health, Faculty of Veterinary Medicine, University Utrecht, Utrecht 3584CL, The Netherland; f.j.c.m.vaneerdenburg@uu.nl; 4 Epidemiology and Poultry Health Care, Department of Farm Animal Health, Faculty of Veterinary Medicine, University Utrecht, Utrecht 3584CL, The Netherlands; f.c.velkers@uu.nl; 5 Animal Welfare and Laboratory Animal Science, Department Animals in Science and Society, Faculty of Veterinary Medicine, University Utrecht, Utrecht 3508TD, The Netherlands; s.s.arndt@uu.nl

**Keywords:** animal welfare, mutilating procedures, housing conditions, management practices

## Abstract

**Simple summary:**

Intensive farming systems are confronted with a number of animal welfare issues such as injuries from horns in cattle and feather pecking in poultry. To solve these problems, mutilating procedures, such as dehorning in cattle and goats and beak trimming in laying hens, are applied routinely. These and other procedures such as early maternal separation, overcrowding, and barren housing conditions impair animal welfare. Scientific underpinning of the efficacy of these interventions and management practices is poor. We advocate that all stakeholders, in particular animal scientists and veterinarians, take the lead in evaluating common, putative mutilating and welfare reducing procedures and management practices to develop better, scientifically supported alternatives, focused on adaptation of the environment to the animals, to ensure uncompromised animal welfare.

**Abstract:**

A number of mutilating procedures, such as dehorning in cattle and goats and beak trimming in laying hens, are common in farm animal husbandry systems in an attempt to prevent or solve problems, such as injuries from horns or feather pecking. These procedures and other practices, such as early maternal separation, overcrowding, and barren housing conditions, raise concerns about animal welfare. Efforts to ensure or improve animal welfare involve adapting the animal to its environment, i.e., by selective breeding (e.g., by selecting “robust” animals) adapting the environment to the animal (e.g., by developing social housing systems in which aggressive encounters are reduced to a minimum), or both. We propose adapting the environment to the animals by improving management practices and housing conditions, and by abandoning mutilating procedures. This approach requires the active involvement of all stakeholders: veterinarians and animal scientists, the industrial farming sector, the food processing and supply chain, and consumers of animal-derived products. Although scientific evidence about the welfare effects of current practices in farming such as mutilating procedures, management practices, and housing conditions is steadily growing, the gain in knowledge needs a boost through more scientific research. Considering the huge number of animals whose welfare is affected, all possible effort must be made to improve their welfare as quickly as possible in order to ban welfare-compromising procedures and practices as soon as possible.

## 1. Introduction

Mutilating procedures [1,2], certain animal management practices (e.g., [3]), and housing conditions (e.g., [4]) may impair animal welfare. Many of these practices and conditions, which have often developed on the basis of on-farm experience, are intended to prevent or solve problems inherent to industrial farming, such as difficulties with handling animals (e.g., disbudding in cows, sheep, and goats) or production losses (e.g., farrowing crates to prevent crushing of piglets). While a number of these practices are claimed to have a positive effect on animal welfare and may solve particular welfare problems (beak trimming—less feather pecking; tail amputation—less tail biting) [5], systematic scientific appraisal is needed to check whether the proposed solutions serve the intended goal and how they affect animal welfare.

This article describes common mutilating procedures, management practices, and housing conditions that may affect farm animal welfare. Furthermore, the role of the veterinarian, who operates at the front line of animal health and welfare, is discussed with respect to improving welfare and directing future developments in farm animal management. Steps to identify and replace interventions and procedures that compromise welfare are suggested.

Although there is some evidence of the welfare effects of a number of the abovementioned procedures and practices, continued, targeted research to investigate which management and breeding practices minimize impacts on animal welfare, while recognizing the reality of the business task faced by food producers, is important and necessary [6]. It is the duty of veterinarians and animal scientists to provide this evidence [7].

## 2. Concepts of Animal Welfare

Animal welfare relates to more than merely the physical health of an animal. While there are numerous concepts of animal welfare (e.g., [8,9,10,11,12,13,14,15]), the current understanding is perhaps best summarized by Webster ([16], p. 117) as follows: “There is now broad agreement amongst academics and real people that the welfare of a sentient animal is defined by how well it feels; how well it is able to cope with the physical and emotional challenges to which it is exposed”. Indeed, current welfare concepts put emphasis on the perception of the animal itself (e.g., [11,14,17,18,19,20]). The idea that animals have evolved in adaptation to their environment, so as to optimize their ability to adapt to changes within that environment through the expression of a variety of physiological and/or behavioral responses, was first applied to animal welfare in the 1990s (see, for example, [21]). In this view, an animal’s welfare is not at risk as long as the animal is able to meet environmental challenges [11,18,19], elaborated by, among others in [22,23].

Even though there is broad agreement on how to approach the concept of animal welfare, the definition of markers or measures of welfare and how they should be evaluated remains a major challenge. In recent years, a number of studies have attempted to develop and validate tools and protocols to assess animal welfare (e.g., [5,24,25,26]), such as the Welfare Quality^®^ project [27]. Recently, a new initiative, Animal Welfare Indicators (AWIN), financed by the EU VII Framework Program (FP7-KBBE-2010-4), has been initiated to address animal welfare indicators, with special emphasis on sheep, goats, horses, donkeys, and turkeys. The aim of this initiative, as it was for the Welfare Quality^®^ project [27], is developing protocols for assessing animal welfare, studying the animal welfare implications of diseases and pain, examining the effects of different prenatal social environments, social dynamics, and prenatal handling methods on the development and welfare of the offspring, and, finally, “ensuring that the very best scientific information on animal welfare is easily available to stakeholders and the public” [28].

Mellor recently introduced the concept of “The Five Domains”, seeking to assess the impact of the social and physical environment on the affective state of an animal [15]. Webster [29] discussed the relative validity and utility of this approach in comparison to “The Five Freedoms” as described by the Farm Animal Welfare Council [30] and based on the seminal work of the Brambell Committee [31].

The “Five Domains” indeed provide a useful tool to evaluate “particular physical/functional disruptions and imbalances, as well as restrictions on behavioral expression, and then to identify the specific negative affects each disruption, imbalance or restriction would be likely to generate” ([15], p. 8). However, in our opinion, concepts of welfare should also take the dynamics of the individual’s interaction with its environment over time into consideration. Any attempt to assess animal welfare thus needs to consider changes in measures over time. Thus, the assessment of welfare should focus on whether or not the animal has the freedom and capacity to react appropriately (i.e., adaptively) to environmental challenges.

This approach forms the basis of the concept of animal welfare as introduced by Ohl & van der Staay:

“An individual is in a positive welfare state when it has the freedom adequately to react to

- hunger, thirst or incorrect food;
- thermal and physical discomfort;
- injuries or diseases;
- fear and chronic stress; and thus,
- the freedom to display normal behavioural patterns that allow the animal to adapt to the demands of the prevailing environmental circumstances and enable it to reach a state that it perceives as positive.” ([19], p. 17).

**Figure 1:**
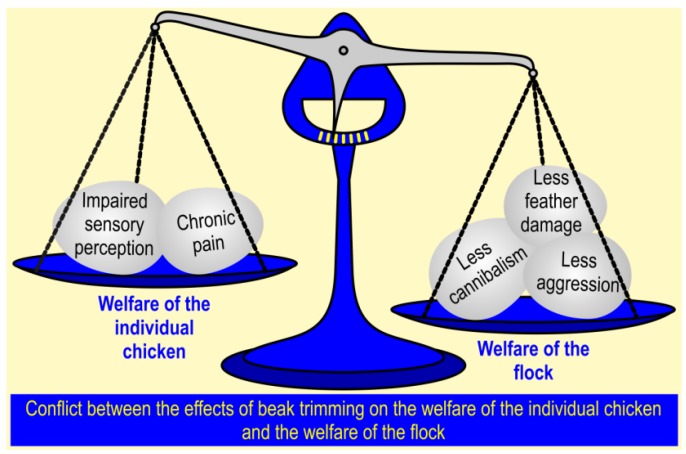
Beak trimming in chickens. This procedure reduces the consequences, but not the incidence, of severe feather pecking. Although it causes discomfort in the individual chicken, the flock may profit from this intervention because the consequences of feather pecking are generally less severe [33,37]. The general question thus is whether the harm inflicted on the individual outweighs the benefits for the individual and/or the group [2]. This figure shows, as a hypothetical example, the evaluation concluding that the effects of beak trimming are favorable for the welfare of the flock. Depending on a different weighing of the arguments, the conclusion may be that prophylactic beak trimming is unacceptable. For example, in breeds with a very low incidence of feather pecking, trying to reduce the suffering of some of the birds by mutilating all of them seems completely unacceptable from a purely animal welfare perspective, whereas in breeds with a very high incidence of feather pecking, beak trimming might be considered more acceptable. Alternative solutions to beak trimming must be taken into account before even considering the use of mutilating procedures.

In the light of this concept, we need to ask whether current practices and procedures affect the option and capacity of farm animals to react appropriately, and to what extent. It is of outmost importance to search for adequate read-out parameters regarding adaptive capacity and to avoid primarily economically driven judgements. For example, beak-trimmed hens might eat and produce eggs. However, this does not indicate a positive emotional state and the animals might permanently suffer from impaired sensory perception and pain (see Figure 1). To induce positive welfare, adaptive processes must lead to a state that an animal itself perceives as positive [19].

## 3. Procedures, Management Practices, and Housing Conditions That Affect Farm Animal Welfare

### 3.1. Adapting the Animal to Its Environment

Many procedures currently used in the production of animal-derived products (e.g., meat, leather, milk, and eggs) were introduced to counteract problems that emerged when livestock farming became intensified, such as tail docking in fattening pigs in response to tail biting [32], or beak trimming in response to feather pecking in laying hens [33,34]. Other procedures were introduced to improve the handling of animals (e.g., dehorning of cows, gestation crates for sows), or in response to consumer demands (e.g., castration of pigs to avoid boar taint [35]). Thus, in industrial animal farming animals are altered or “adapted” to meet the constraints caused by their housing conditions and the management practices used [36].

### 3.2. Mutilating Procedures

The aims of mutilating procedures are manifold, one of them being to “help” animals adapt to their environment. When discussing the welfare aspects of these procedures, it is important to distinguish between the welfare of the individual animal and the group as a whole. Consider the examples of beak-trimming in chicken [33,37] (see Figure 1), and the clipping of the canine teeth of pigs. Clipping causes discomfort to the individual pig but is thought to be beneficial to the group—theoretically, it prevents mammary injuries in sows and severe injury during fights with pen mates. However, so far there is no evidence that tooth clipping provides the envisaged benefit for the group [38,39,40]. There is an urgent need for such information, to determine whether benefits for the group outweigh the harm inflicted on the individual animal. Prophylactic mutilating procedures might be performed with the intention to reduce welfare risks. These risks, however, need to be assessed first, regarding their degree of impact (are all animals in the flock affected?), duration, intensity, and probability, which needs to be taken into consideration when determining whether the benefits for the group outweigh the suffering of individuals. From an animal welfare as well as a moral perspective, it would be unacceptable to perform ‘unnecessary’ mutilations on individual animals.

An overview of mutilating procedures, management practices, and housing conditions is provided in Table 1. The mutilating interventions in Table 1 (A) have already been addressed in a large number of scientific papers. We want to direct attention now to the housing and management practices causing predominantly physical, psychological, and emotional discomfort, and consequently we shall expand on entries in Table 1 (B, C). Not all points raised in these two parts of the table have already gained sufficient scientific attention. Generally speaking, the gaps in scientific knowledge will be closed as soon as there are scientifically valid translations to farming practices that have been proved to improve animal welfare.

### 3.3. Individual Housing of Social Animals and Confinement

**Cattle:** Calves are usually separated from the cow and housed individually shortly after birth. We will discuss the practice of housing newborn calves in hutches or igloos in detail in the paragraph about maternal separation.

**Pigs:** In industrial pig farming it is common practice to house farrowing sows in crates. There is some evidence that sows are able to cope with extremely adverse housing conditions, such as long-lasting tethering during gestations, farrowing, and suckling, a practice that is no longer permitted in The Netherlands (e.g., [41,42]). However, even if sows are able to cope with adverse housing conditions, their welfare may be impaired if the required adaptability of the animal is high and approaches the limits of adaptability of the animal [19]. Farrowing crates severely restrict freedom of movement and do not allow normal postural adjustments [43]. Farrowing crates are used in order to prevent piglets being crushed. The challenge is to find alternative systems, giving more freedom to the sow while maintaining a high piglet survival rate and taking the welfare of the sow and her piglets into account (e.g., [44,45]).

### 3.4. Lighting Regimens—Artificial Lighting

**Pigs**: Pigs are kept under a large range of light–dark periods and light intensities. Using operant preference tests, pigs are willing to work to switch on a light [46,47], but not to get access to darkness [46]. In both studies, pigs appeared to prefer lower light intensities. Pigs are sometimes kept in semidarkness in order to prevent aggressive behavior. However, this light condition impairs explorative behavior. The welfare of the animals might be improved by an appropriate light regimen, as found, for example, by Martelli et al. [48].

**Chickens**: A large variety of photoperiods are applied in broiler production, from continuous light to intermittent schedules with a number of successive light on–light off periods [49], based on the assumption that long light exposure will lead to maximal growth. Whereas their effects on performance have been addressed (e.g., [50]), the welfare consequences of these schedules have not yet been well established (a situation nearly unchanged since a review [51] about this topic, written 20 years ago). An optimized light–dark regimen combined with optimal stocking density may improve the health of broilers and positively affect their welfare, e.g., by reducing chronic fear [52]. Very low light intensities (1 lx) may impair welfare [53].

### 3.5. Feed and Water Restriction

So far, there is no detailed legislation regarding water supply for farm animals. However, scientific evidence for the effects of water restriction is constantly growing:

**Pigs:** Wet-fed pigs are generally water-restricted in order to reduce the total volume of slurry effluents. This could, however, negatively impact pig welfare. As Vermeer et al. [54] and Nannoni et al. [55] showed, wet-fed pigs are still motivated to obtain additional fresh water from drinkers.

**Chickens:** Feed restriction is routinely applied in broiler breeders in order to control growth and body mass [56]. These broiler breeders show high motivation to gain access to a foraging area, even if this area does not contain food [56]. They also show deficits in learning and memory: they perform worse in a Y-maze task than mildly food restricted control animals [57]. Indications of hunger are persistent and not reduced by varying feeding schedules (i.e., scattering feed once versus twice per day [58]). Food restriction also affects social interactions, as restricted male broilers show more aggressive behavior than male broilers fed ad libitum [59].

Water restriction, although forbidden, sometimes is applied in broilers to reduce wet litter, a main cause of footpad dermatitis [60]. Chickens may undergo water restriction resulting from adverse social interactions (e.g., chickens lower in the hierarchy being denied access to a limited water supply) or as a consequence of illness or injury preventing a chicken from accessing water. Layer hens deprived of water show a strong motivation to access water as well as remaining in the vicinity of drinking points after 12 h of water deprivation [61]. Increasing the number of drinkers can mitigate this problem.

### 3.6. Overcrowding/Social Instability

**Pigs:** Overcrowding in pigs forms a potential risk for the health and therefore the welfare of the animals. Enteric and respiratory disease due to reduced hygiene and air quality when stocking densities are high might occur, as, for example, discussed within the Scientific Opinion of the Panel on Animal Health and Welfare on a request from the Commission on Animal health and welfare in fattening pigs in relation to housing and husbandry [62]. Overcrowding might furthermore lead to problems like leg weakness and a higher prevalence for some claw lesions, as discussed e.g., by Jørgensen [63], and could make aggressive encounters more likely.

**Chickens:** Large stables, housing thousands of chickens, challenge and exceed the animals’ capacity to individually recognize their stable- or pen-mates [64]. This may hamper the establishment of a hierarchy, which safeguards social stability in natural groups. However, unexpectedly, in very large groups aggression decreases [65], i.e., “non-aggressive strategies may be more efficient than fighting for resources when group size is large” ([65], p. 198).

On the other hand, it has been reported that overcrowding in broilers increases serum corticosterone levels [66]. The physical wellbeing of broilers in overcrowded conditions is compromised, as seen in increased injuries, reduced locomotion, and higher mortality rates [67] and in stress parameters such as an increased heterophil/lymphocyte ratio, which is considered to be a reliable stress marker [50].

However, in a large scale study by Dawkins and colleagues, other factors, such as the quality of stockmanship, temperature, and humidity in the stable appeared to affect the welfare of broilers more than stocking density (expect for very high stocking densities [68]). Determining an optimal group size and crowding density may thus contribute to improving welfare in broilers [69] and other farm animal species.

European legislations for adult laying hens dictate that hens in enriched cages must have at least 750 cm^2^ of cage area per hen and for hens in alternative systems a maximum stocking density of nine hens per m^2^ is allowed. However, there is no legislation in place for stocking densities during the rearing period of laying hens from hatching until approximately 18 weeks of age, which is a substantial part of the life of the laying hen [70]. Evidence-based legislation is urgently needed for the stocking densities during the rearing period that safeguard animal welfare.

### 3.7. Repeated Mixing

In general, separation from the group, regrouping (mixing), and (re-)introduction to their own or a new group in social animals such as cattle, sheep, goats, pigs, and chickens can negatively affect welfare. In particular, these practices interfere with social recognition, a cornerstone of group cohesiveness and group structure [71]. These species are able to individually recognize a large number of group members (chicken: [72], cattle: [73], goats: [74], sheep: [75], pigs: [76]), although very large groups or flocks may exceed their recognition capacity, which hampers establishing a hierarchy and induces social instability.

**Goats:** Patt and colleagues showed that separation of goats from and re-introduction in the group [77], and introduction of goats into established groups [78], is stressful. One measure to reduce the effects of separation and re-introduction may be to enable the (separated) goat to still hear and smell the other goats [77].

**Pigs:** In pigs, repeated mixing is common practice, sometimes driven by the desire to maintain very homogeneous groups per pen. After weaning, sows are returned to group housing. The weaned piglets are transferred to and usually mixed with piglets from other litters, which may lead to aggression [79]. Adverse effects of mixing may increase as a consequence of the intended abandoning of castrating male pigs [80]. Entire male pigs show a higher level of aggression than castrated pigs [81]. However, socializing pigs early [82] and keeping them in intact (sibling) groups until slaughter can significantly reduce aggression and stress [81,82] and, consequently, improve pig welfare.

A study by Fels and colleagues [83] showed that pigs are able to establish a linear (groups of six pigs) or quasi-linear (groups with 12 pigs) social hierarchy within three to four days after mixing, irrespective of whether groups were homo- or heterogeneous. Larger groups may retard or even hamper social hierarchy formation, maintain aggression, and impair welfare.

**Chickens:** Social mixing occurs in both layer and broiler chickens as they are transferred from one farm type to another: from a hatchery to a rearing farm, and then to a laying farm, in the case of layers; and from a hatchery to a growing farm in the case of broilers. Mixing unfamiliar chickens may provide an unwanted source of stress, as social hierarchies need to be re-established and group stability re-formed. The question of social mixing in chickens after hatching or in transport between farms has yet to be examined in controlled experiments. However, repeated social disruption has been shown to affect serotonergic and dopaminergic systems in the brain in layer hens [84]. Social mixing during the various phases of poultry farming may well be a stressor, though further research is needed to establish whether or not this is the case. Recent innovations in broiler housing may be able to reduce mixing in broiler chickens, such as the ‘on-farm hatching concepts’ that combine the hatching and brooding phase. This avoids mixing between hatching and rearing, increases hatchability, and reduces mortality in broiler chicks [85]. Furthermore, it provides the chicks with the opportunity to eat and drink directly after hatching.

### 3.8. Individual Housing (of Social Animals)

**Pigs:** Boars are housed individually in many sow stables to detect estrous expression in the sows [86]. These boars are usually kept in a small confinement near the group-housed sows. The boars can see and smell the neighboring sows, but cannot contact them. To our knowledge, the welfare consequences of this housing practice on the individually housed boars have not yet been investigated scientifically.

### 3.9. Early Maternal Separation

**Cattle:** In most intensive farming systems, dairy calves are separated from their mother in the period immediately after birth to a few days after birth and housed individually indoors in isolated barns, or outdoors, in hutches or igloos [87]. This practice is controversial [3]. The main reason for this practice is to reduce the risk of transferring infectious diseases to the newborns. The consequences of this practice for the welfare of cows and calves have been addressed in a number of publications. It appears that the stress response in both cow and calf is minimal if bonding is forestalled. Investigating the effects of separation starting 6 h, one day, four days, or two weeks after birth, Weary and colleagues [88,89] found that the longer the calf stayed with the cow before separation, the stronger the behavioral responses of both were. On the other hand, socializing the calf may profit from staying with the dam, preferentially in a group. This is one of the pillars of the “family herd” concept [90].

**Goats**: In goat farming, kids are usually separated from the doe within a few hours after birth, after the kids have ingested colostrum. To our knowledge, the putative welfare implications of early maternal separation for doe and kids have not yet been addressed scientifically.

**Chickens**, whether layers or broilers, in industrial farming will never see an adult chicken, but are usually hatched, reared, and kept in same-age (peer) groups. Under (semi-)natural conditions, chicks gain information about palatable food from their mother. This information may be relevant at least for chickens that have access to an outdoor run. In industrial farming, chicks are not given the opportunity to learn from an adult. The implications of this practice have not yet been studied systematically.

### 3.10. Weaning

**Pigs:** Pigs are usually weaned, i.e., withdrawn from milk supply by the sow and introduced to solid adult feed, at the relatively young age of four weeks. A less abrupt weaning at a higher age may improve the welfare of sow and piglets. During (an extended) pre-weaning period, piglets may learn from the sow to eat novel foods and to increase their intake of solid food. Interaction with the sow may also help reduce the development of damaging behaviors and increase play behavior after weaning [91]. Another measure described to improve welfare in piglets is pre-weaning socialization, in which barriers between farrowing pens are removed, allowing pre-weaning piglets from different litters to interact [92,93].

**Cattle/sheep:** Due to the common practice of separating the dairy calf from its mother shortly after birth, the weaning process predominantly consists of the transition to solid feed. In beef cattle and sheep, a two-stage weaning procedure has been developed in which the calf/lamb is prevented from drinking milk from its mother for a period of time before separation from the mother. In calves, a nose flap prevents the calf from drinking the cow’s milk [94,95]; in sheep, the lamb is prevented from drinking the ewe’s milk by covering the ewe’s udder with a net [96]. Two-stage weaning may cause less stress and distress than the usual one-stage weaning process, although more research may be needed as the results are not yet unequivocal [94]. More research is also needed regarding alternative methods, e.g., Enriquez et al. [97].

### 3.11. Barren Environment

**Pigs:** The behavioral pattern of domesticated pigs is highly conserved and consists of the full behavioral repertoire of the wild boar [44,98,99]. Most pigs are kept in barren pens with a concrete and partially slatted floor. In these environments, pigs are usually provided with enrichment material such as chains and bite sticks [100]. However, pigs are highly motivated to root [101,102], a behavior that cannot be executed if the flooring substrate is absent. It has been found that the lack of rooting material directs pigs’ behavior towards pen-mates and increases aggression, ear chewing, licking, and biting pen-mates, It also increased belly nosing, tail biting, and play fighting [103]. Provision of suited rooting material thus may help to control undesirable behavior in pigs.

Domesticated sows engage in complex nest-building behavior starting approximately 1½ to ½ days before farrowing [104,105]. In barren farrowing pens that do not provide substrate suited for nest building, this will translate to restless, abnormal behaviors that are likely redirected nesting behavior, whereas animals provided with substrate will build a nest [106]. Depriving pre-parturient sows of nest building impairs their welfare because it limits expression of their behavioral needs [104]. Moreover, sows in farrowing crates appear to engage in less maternal behavior than loose sows [107]. Considering the biological significance of nest-building behavior in sows, the farrowing systems should facilitate its expression [44]. Post-partum, crushing of piglets is a serious welfare problem. Whereas under (semi-) natural conditions, the sow will perform a behavioral sequence that will guide the piglets away from the area where the sow lies down, the lack of space in most farrowing crates does not allow this behavior, and the loss of piglets increases [108].

**Chickens** are reared in environments that can vary vastly, ranging from battery cages (currently banned in the EU but still standard practice in many other regions of the world) to furnished cages, aviary systems, and free-ranging. The complexity of the environment does seem to influence chicken welfare, as heterphil:lympocyte ratios, a common physiological measure of stress, are decreased in chickens raised in furnished compared to barren cages [109].

## 4. Improving Animal Welfare

**Figure 2:**
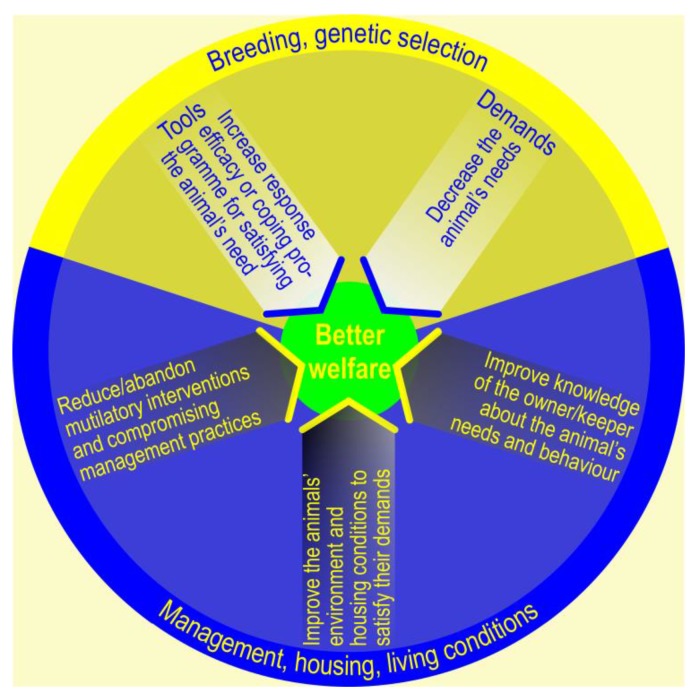
Strategies for improving animal welfare (inspired by [110]). Improving the animal’s tools (abilities) to cope efficiently with its environment or decreasing the animal’s demands (needs) via breeding programs and genetic selection are goals of ongoing activities. In particular, the strategy of reducing the animal’s demands by using genetic selection procedures is subject to ethical discussions [112,113]. Alternatively, one may reduce or abandon mutilating procedures, improve the animal’s housing conditions, and improve the knowledge of owners/keepers/stockmen with respect to the animal’s needs and demands. In particular, with respect to management, housing, and living conditions, animal welfare scientists and veterinarians should deploy their expertise, give (un)solicited advice, and assume an active role in the welfare discussion (see also Figure 3).

Kanis and colleagues [110] distinguished two main strategies for improving animal welfare: (1) selective breeding for desired traits (e.g., breeding for less expression of harmful social behaviors like aggression and savaging in pigs [111]) and (2) improvement of management routines and housing conditions. In general, the aim of breeding and genetic selection may be to decrease an animal’s needs and/or to increase its abilities in an attempt to reduce possible negative consequences for welfare. Selective breeding, in combination with improved management practices and stockmanship and more appropriate housing, together with the abandonment of mutilating procedures and other practices that compromise animal welfare, is expected to improve and ensure farm animal welfare (see Figure 2). Animal scientists and veterinarians trained as welfare experts are highly suited to steer and supervise these changes, due to their expert knowledge of animal welfare, in addition to their close contact with farmers.

## 5. Breeding and Selection Programs

The aim of breeding and selection programs is to adapt animals to their environment [118] (see Figure 2), for example by increasing their adaptability [119]. Many traits that are relevant for biological functioning are under genetic control and are expected to respond to genetic selection [120,121]. Recently, Nicol and colleagues noted that “genetic selection and management strategies derived from a fundamental understanding of the basis of feather pecking behavior compare favorably with current practices of beak-trimming and light reduction as potential control methods” ([34], p. 776) Programs have been initiated to control and diminish harmful social behavior, such as feather pecking in poultry and tail biting in pigs, through selective breeding out of the undesired behaviors [111]. Kjaer and colleagues [122,123] and Rodenburg and colleagues [124,125] initiated selective breeding programs for laying hens to control and reduce feather pecking and cannibalism, and other programs focus on selecting out undesired traits such as aggression [125,126]. Over time, these approaches may indeed lead to a better match between the animal and its environment. 

The selection of “robust” farm animals may have a similar effect. Robustness refers to “an increased adaptation to a range of environmental conditions” ([127], p. 343), i.e., an increase and/or restoration of the animal’s coping ability or general adaptability, and improvement of its immune system and general health [128]. However, there is no specific “robustness” trait [127] and this strategy is not undisputed, especially for ethical reasons [112,129]. For example, such selection programs may produce unresponsive animals that do not express pain or discomfort even though they are suffering [112].

We suggest that the causes of undesired traits and behaviors should be investigated as the most relevant starting point for research. Then breeding programs could focus on these causes rather than on eliminating undesired traits and behaviors. As mentioned above, the latter approach may generate other, sometimes more urgent, welfare issues.

While selection programs focusing on susceptibility to stress, vitality, aggression, stereotypies [130,131], and sociality [121,132] may be societally desirable, they may not directly translate into economic advantage to the farmer [132]. However, as society is becoming more aware of, and verbal about, the welfare issues involved in industrial animal farming, these programs may provide an economic advantage in the long run. Veterinarians need to explain the long-term advantages to farmers, basing their arguments on scientific evidence.

## 6. Enabling the Animal to Cope with Its Environment

A different approach from altering the animal that deserves more attention, and scientific substantiation, is to help animals to cope with their environment. A first step might be to create rearing conditions that better “prepare” animals for the environment in which they will be kept in later stages of their life. For example, chickens that are kept in aviaries should be reared in an environment that provides opportunities to learn to use perches [133]. Another option is to train animals to comply with management and husbandry procedures [134,135], such as training pigs to use automatic feeders [136].

## 7. Adapting the Environment to the Animal

### 7.1. Housing Conditions

Another approach is to adapt the animal’s environment to meet its needs and to comply with animal welfare demands. This strategy was suggested more than 3½ decades ago by Faure [137] and has since been discussed by others (e.g., [138]). The artificial housing environment of production animals can be extremely constraining in terms of the animals’ freedom to interact with their environment appropriately, for example because of restricted space, overcrowding, lack of retreat possibilities, high levels of noise, ultra- or infrasonic sound, light characteristics (level, spectrum, frequency), aversive routine husbandry, or abnormal social group compositions [139,140] (see Table 1 (B, C)). Consequently, “the farming system must be designed to fit the animal” ([141], p. 1481) and must ensure that animals can function properly.

The European Union took this approach when it made the social housing of gestating sows compulsory in an attempt to improve sow welfare [142]. The ban on gestation crates came into force in January 2013. While this system, with gestation pens or pens with electronic sow feeders, is undoubtedly more appropriate to the animal’s needs than individual housing in gestation crates, aggressive encounters and injuries frequently occur in social housing (e.g., [143,144]) and welfare might be at risk. There is thus an urgent need to develop social housing systems in which aggressive encounters are reduced to a minimum (such as, for example, the comfort class concept for pigs: [145]).

It is not sufficient to ban practices, such as specific housing systems, without providing an alternative that constitutes a proper match between the animal’s needs and its abilities to interact with an environment and the actual environmental demands and constraints. The improvement of housing conditions and systems to monitor welfare [27,146,147] are steps in the right direction to improving animal welfare by improving the animals’ environment.

### 7.2. Management Practices

Management practices can have a tremendous effect on animal welfare. For example, good stockmanship and non-aversive handling methods can make animals less fearful and more productive [148,149]. Some practices are relatively easy to apply, such as well-maintained bedding in cubicles for dairy cows, which reduces lameness and swollen hocks [150,151,152,153]. The monitoring of lameness, body condition, and flight zone by veterinarians can help improve the welfare of animals on farms (see also [154]).

Veterinarians working in the field need to prevent obvious abuse and appropriate advice might reduce the incidence of lameness on dairy farms, but in many cases it is difficult to convince farmers to implement management changes that improve animal welfare [155]. This requires good interpersonal skills on the part of veterinarians so that they can explain to farmers the benefits of implementing management practices that improve the welfare of their animals. Veterinary education needs to prepare veterinarians for this role.

New concepts for the management and housing of farm animals have been developed, such as the “Rondeel”, a round housing system for laying hens that consists of different wedges in which the animals are raised under conditions designed to fulfil the natural needs of the animals [156], and the “Comfort Class” concept for fattening pigs, [145]. “Comfort Class” is defined as a specific minimal level of husbandry conditions of animals, at which the ability of animals to meet their needs is not compromised by husbandry conditions” ([145], p. 172). However, the example of the “Rondeel” shows that years may pass before ideas and concepts are put into practice [156,157]. It is inevitable that adjustments are necessary when concepts are put into practice, but these modifications need to be evaluated scientifically to avoid basing modifications on gut feelings or common practice. Despite the best intentions, changes that are not evidence-based may impair rather than increase animal welfare.

## 8. Where to Go from Here?

Fundamental research into animal behavior and welfare is poorly funded, probably because it does not provide instant solutions for welfare problems as perceived by society. Yet, basic research is urgently needed to (re-)evaluate the effects of approaches to improve animal welfare, be it through improving the ability of an animal to adapt to its environment, by modifying the environment to better meet the needs of an animal, or by a combination of these approaches. Attempts to resolve serious mismatches between animal and environment are often made on a trial-and-error basis, and rarely shed light on the factors and processes underlying the problem. Unfortunately, many commonly used procedures to improve animal housing and management actually have adverse effects on the animal (see Table 1).

In order to assess an animal’s welfare state, it is first necessary to have a thorough knowledge of the animal’s normal behavioral repertoire and its behavioral needs and abilities. This will help farmers and stock keepers and veterinarians to identify constraints to an animal’s needs and its ability to express natural behavior, constraints that may compromise the animal’s welfare [158]. As early as 1978, Kilgour pointed to the need to obtain this basic information: “The ethogram of farm animals should have a high priority for current animal science goals” ([141], p. 1481). Unfortunately, comprehensive ethograms are still not available for farm animals, although ethograms for specific behavioral domains have been published (e.g., [159,160,161]). The behavior of the wild ancestor, or of feral conspecifics, is sometimes used to describe (part of) the behavioral repertoire of domesticated animals [162]. These behaviors are considered to represent the “normal” repertoire of a species. However, this view is based on a number of assumptions [163], which have not yet been sufficiently corroborated scientifically and which can be translated into the following questions:

- Is this “normal” behavior indeed unaltered by domestication?
- What are the effects of (heavy) selection on performance characteristics (e.g., high yield)?
- Does the wild ancestor, or a population that expresses the full pre-domestication genome, exist, and is it available for research?

**Figure 3:**
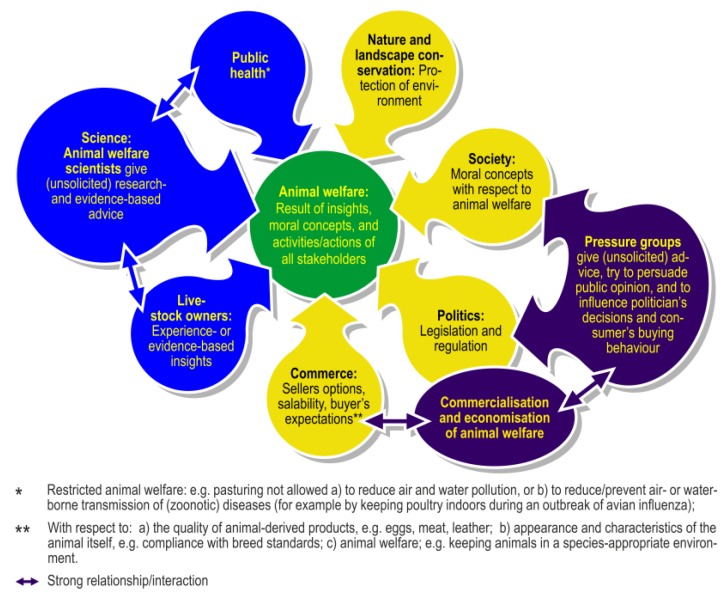
Stakeholders in animal welfare. Animal welfare is the result of (scientific) insights, of moral concepts of scientists and society at large [114,115,116], and of the activities and actions deployed by all stakeholders. Animal welfare scientists have an obligation to perform scientific research on animal health and welfare, to take action if animal welfare is compromised, and to give direction to future developments. Politicians are sensitive to the opinions and sentiments of pressure groups and society at large and may amend legislation and regulations. In parallel, the commercial value of animal welfare is increasingly being recognized and creates added value for many animal-derived products (e.g., [117]) and services. Note: Veterinarians are involved in ensuring public health, and they are the primary contact persons about animal health/welfare with livestock/animal owners.

The current lack of comprehensive ethograms is a serious obstacle to evaluating farm animal welfare, taking steps to improve welfare, and assessing the effects of these actions. In order to fully understand whether behaviors observed are normal or abnormal/harmful, it is imperative to have a full understanding of the behavioral repertoire of a species. This full description of the behavior of a species, in the form of a complete ethogram, is currently lacking for farm animals in general, despite the seminal work of describing, for example, the behavior of pigs in their natural environment [99]. Last but not least, the definition of welfare should integrate scientific insights and moral concepts [16,19], such as those of animal scientists and society at large, e.g., [164,165] (see Figure 3). Quantifiable measures or indicators, for example behavioral measures, are necessary to determine the current welfare state of an animal, to define a target value (based on knowledge about the animal’s needs and abilities, i.e., ability to cope), and to monitor progress. Such target values should be updated to reflect new knowledge and insights into animal welfare and the role of the stakeholders in the welfare discussion [166]. The accomplished animal welfare is the result of insights, moral concepts, and activities/actions of all stakeholders (see Figure 3) (see, e.g., [167,168,169,170,171]).

In the final step, the translation of welfare concepts into farm animal practice, the veterinarian is at the forefront, directly advising farmers, steering and supervising changes. This key position makes it necessary to equip veterinarians with fundamental expertise, especially in animal behavior. This consideration has recently been adopted by the faculty of Veterinary Medicine at Utrecht University, The Netherlands, by establishing a curricular focus on animal behavior [172].

## 9. The Commercialization of Animal Welfare

The commercial potential of animal welfare has been increasingly noticed in recent years. Commercialization and economization may be achieved by awarding quality labels and selling labeled products at a higher price than unlabeled products. For example, the Dutch Animal Welfare Association (Dierenbescherming) awards one, two, or three “stars” of their “Beter leven keurmerk” (“Better Life Quality Mark”) to animal-derived products from producers who fulfill the minimum requirements with respect to housing and managing the animals (See for costs of this quality label for the participating companies: [173]). Quality label awarding organizations such as the Dutch Animal Welfare Association generate income with this quality mark (e.g., in 2013: 748,000€ [174], in 2014: 858,000€ [175]). On the other hand, these organizations face considerable costs for controlling and ensuring the welfare level guaranteed by the label.

In a win-win situation—improving animal welfare, gaining money from awarding a welfare quality mark for the awarding organization, and higher prices for these products for the retailers—this may boost the introduction and implementation of measures that improve animal welfare. Selling welfare quality labeled animal-derived products will not only justify higher prices, but may also increase the retailer’s reputation.

It can be a time-consuming and difficult process to reach an agreement between different stakeholders to improve animal welfare. However, once an agreement has been reached and implemented, it may be extremely difficult to put negotiations about new steps to further improve animal welfare on the agenda. It remains to be seen whether the economization and commercialization of animal welfare improves animal welfare in the long run.

## 10. Conclusions

Farm animals are the largest group of animals kept by humans, and thus the focus of welfare research is likely to stay on farm animals [6]. Procedures that compromise welfare and biological functioning should be identified, abandoned, and replaced by alternatives that do not adversely affect animal welfare. Strategies to meet this goal include selective breeding, improved stockmanship, as well as improving management routines, housing, and rearing conditions. The decision to apply certain management—and especially mutilating procedures—needs to be based on scientific evidence and evaluated against social norms and values. Thus, more research, in particular on the long-term consequences of such procedures, is urgently needed.

The responsibility of humans to safeguard the welfare of domesticated animals is directly associated with the animal’s perceived ability to adapt to its environment. This may be influenced by selection/breeding processes, or by the animal husbandry system to which the animal is exposed. The involvement of many stakeholders in the discussion of farm animal welfare (Figure 3) is an added complication, because these stakeholders may have different perceptions of the importance of animal welfare [171,176] and of the measures needed to improve and ensure welfare. Veterinarians and animal scientists are key players who should have fundamental expertise in animal behavior and welfare as well as ethics [177]. They should closely cooperate [178] and should actively participate in directing future developments [7] in farm animal management and animal housing, with a view to improving animal welfare. In this, they should be guided by their professional codes of conduct and ethics (although these codes may require harmonization [179]) in terms of their role in ensuring and improving farm animal welfare. It remains to be seen whether commercialization will benefit animal welfare.

## Figures and Tables

**Table 1:** Mutilating and damaging interventions and housing/management practices, applied sporadically or routinely, which have the potential to impair farm animal welfare (a summary inspired by [2]).

	Interventions	Cattle	Sheep	Goats	Pigs	Chickens
A. Mutilating interventions causing physical discomfort	Castration	✚		✚	✚✚	
	Docking (tail amputation)				✚✚✚	
	Disbudding	✚✚✚		✚		
	Dehorning		✚			
	Ear notching				✚	
	Ear tagging—wing band—toe slit	✚✚✚	✚✚✚	✚✚✚	✚✚✚	✚
	Teeth clipping				✚	
	Nose ringing	✚				
	Beak trimming					✚✚✚ ^**a**^
	Despurring—toe clipping					✚
	Dubbing					✚
	Caesarean section	✚ ^**b**^				
B. Housing and management practices causing predominantly physical discomfort	Confinement ^**c**^	✚✚✚			✚✚✚	✚
	Lighting regimens—artificial lighting				✚✚	✚✚✚ ^**d**^
	Feed and water restriction				✚ ^**e**^	✚✚✚ ^**f**^
C. Housing and management practices causing predominantly psychological and emotional discomfort	Overcrowding/social instability	✚✚		✚✚	✚✚	✚✚✚
	(Repeated) mixing	✚✚✚	✚	✚✚✚	✚✚✚	✚✚
	Individual housing (of social animals)	✚			✚ ^**g**^	✚
	Early maternal separation	✚✚		✚✚✚	✚✚✚	✚✚✚
	Barren environment			✚ ^**h**^	✚✚✚	✚✚

Estimated occurrence: ✚ In some management systems; ✚✚ in many management systems; ✚✚✚ in virtually all management systems. ^**a**^ In laying hens and in breeders of layers and broilers; ^**b**^ In a considerable percentage of two beef cattle breeds, Verbeterd Rood Bont (“Improved Red and White”) and Belgian Blue, natural calving is nearly impossible, necessitating Caesarean sections. This concerns approximately 15% of the beef cattle stock in the Netherlands; ^**c**^ Calves in hutches or igloos; sows in crates; chickens in cages; ^**d**^ Absence of natural daylight and/or light intensity or regimens that do not facilitate a diurnal rhythm/resting period; ^**e**^ Sows, growing/finishing pigs if fed liquid feed; ^**f**^ Mainly in broiler breeders (to prevent high body weights), in layers to induce molting (infrequent), and water restriction in broilers to reduce wet litter (not allowed); ^**g**^ Boars (breeding); ^**h**^ Housing of dairy goats in deep litter (goats prefer solid and dry surfaces), indoor housing, no pasture. Mutilation is used here for (A) interventions or (B) housing conditions and management methods that produce acute and/or lasting physical discomfort. In (C), housing and management methods are listed that lead to lasting psychological (emotional) discomfort. Of course, physical discomfort will also affect the emotional state, and thus the overall welfare of the animal, adversely. In turn, emotional discomfort can lead to physical discomfort. Note: The occurrence of these procedures has been estimated on the basis of the expert opinion of five veterinarians and two animal scientists of the Department of Farm Animal Health of the Faculty of Veterinary Medicine at the University of Utrecht. The mutilating procedures are those carried out in the EU. This list is not comprehensive and mutilations/suffering caused by unworkmanlike catching, transportation, slaughtering, bad stockmanship, and bad management, such as infrequent culling of suffering animals, infrequent claw trimming, tardy treatment, and housing conditions such as bad climate conditions, flooring or housing that causes injury or lameness, poor cleanliness, leading to dirty animals covered in manure, etc. are not included. They may occur under all housing conditions and management systems. Also, a number of interventions, such as hot or freeze branding and teeth grinding in sheep that are forbidden in the European Union are not on the list. Such interventions, however, may occasionally still be applied in Europe, and routinely in countries outside Europe.
